# Use of leflunomide in patients with chronic hypersensitivity pneumonitis

**DOI:** 10.1186/s12890-020-01227-2

**Published:** 2020-07-21

**Authors:** Sungryong Noh, Ruchi Yadav, Manshi Li, Xiaofeng Wang, Debasis Sahoo, Daniel A. Culver, Aman Pande

**Affiliations:** 1 Respiratory Institute, Cleveland Clinic, 9500 Euclid Ave. /A 90, Cleveland, OH 44195 USA; 2 Lerner Research Institute, Cleveland Clinic, Cleveland, OH USA

**Keywords:** Hypersensitivity pneumonitis, Immunosuppressant, Leflunomide, Fibrosis, Pulmonary function

## Abstract

**Background:**

Prednisone has been shown to reverse lung function declines in hypersensitivity pneumonitis patients without established fibrosis. Second line immunosuppressants like azathioprine and mycophenolate mofetil have a steroid sparing effect and improve DLCO. There is no published literature on the use of leflunomide in such patients.

**Methods:**

We reviewed our experience with leflunomide for treatment of chronic hypersensitivity pneumonitis in 40 patients. We stratified patients according to the presence or absence of significant (> 20%) fibrosis. We studied the effect of leflunomide on FVC and DLCO trajectory and reported the changes at 12 months.

**Results:**

Treatment with leflunomide tended to improve the estimated FVC slope from 0.18 ± 1.90% (SEM) of predicted per year to 4.62 ± 1.65% of predicted (NS, *p* = 0.118). It significantly improved the FVC at 12 months of treatment by 4.4% of predicted (*p* = 0.02). DLCO continued to increase at 1.45 ± 1.44% (SEM) of predicted per year. Non-fibrotic cHP patients had the largest gain in pulmonary function. Their FVC increased by 8.3% (*p* = 0.001) and DLCO by 4.8% (*p* = 0.011). Patients with fibrotic cHP did not improve. Leflunomide treatment was associated with significant gastrointestinal and other adverse effects leading 40% of patients to discontinue therapy. It had a significant steroid sparing effect with half the patients weaned off prednisone entirely.

**Conclusions:**

Leflunomide appears to be a fairly well tolerated steroid sparing immunosuppressant that improves pulmonary function in cHP patients. It is most effective in patients without significant fibrosis.

## Background

Hypersensitivity pneumonitis (HP) is an interstitial lung disease (ILD), characterized by immune-mediated inflammation and fibrosis resulting from inhalational exposure to inciting antigens [1, 2]. It can be classified into acute/inflammatory and chronic/fibrotic phenotypes based on clinical, radiological and pathologic features [3]. HP is thought to be mediated by T lymphocytes via Th1/Th2 and Th17 immune response pathways which are associated with granulomatous inflammation and fibrosis [2]. Patients with HP usually present with progressive dyspnea on exertion, cough, fatigue and malaise. Left untreated, some may develop diffuse lung fibrosis [2, 4].

Treatment of HP has traditionally involved exposure avoidance if the inciting antigen is identifiable, and pharmacotherapy with corticosteroids or other immunosuppressive agents [3]. Corticosteroids are often used as first line treatment, although they may confer no long term benefit [5]. Immunosuppressive agents such as azathioprine (AZA) or mycophenolate mofetil (MMF) have been shown to favorably influence pulmonary function in patients with chronic HP [6, 7].

Leflunomide (LEF) is an anti-inflammatory and immunomodulatory agent that has been approved by the Food and Drug Administration for the treatment of rheumatoid arthritis (RA). It inhibits de novo pyrimidine synthesis in activated lymphocytes, suppressing them from initiating inflammatory processes [8, 9]. We have been prescribing LEF in some of our patients with progressive HP based on this mechanism of action, presumed benefit and our anecdotal experience.

In this study, we evaluated the tolerability and effectiveness of LEF in patients with cHP by longitudinal analysis of pulmonary function changes. We hypothesized that LEF would be an alternative steroid sparing immunomodulatory drug for treating cHP with a beneficial effect on pulmonary function.

## Methods

A retrospective review was performed utilizing electronic medical records at the Cleveland Clinic (Ohio, USA). A search was performed for patients with the ICD Code for HP (ICD-9495.8 or ICD-10 J67/J67.8/J67.9) and prescriptions of LEF from 2002 to 2018. The diagnosis of cHP had been confirmed through multidisciplinary discussion with our dedicated ILD group. Patients were included if they received treatment with LEF and had at least one pulmonary function test before and after the initiation of LEF. Patients who had follow-up for at least 3 months while on treatment were included for analysis of effectiveness. LEF was added to or replaced other immunosuppressive agents at the discretion of the prescribing physician. Our thoracic radiologist (R. Yadav) reviewed the imaging studies (CT or HRCT), done within 12 months of LEF initiation to evaluate the degree of fibrosis. CT features of reticulation, traction bronchiectasis, traction bronchiolectasis, architectural distortion and honeycombing were used to define fibrosis. The presence of ground-glass, centrilobular nodules and mosaic attenuation suggested inflammatory/non-fibrotic HP. A visual assessment of the extent of fibrosis was performed, and the population was divided into patients with no/mild fibrosis (less than 20%) and significant fibrosis (over 20%).

### Statistical analysis

Patient data were reported in the form of means and standard deviations for continuous variables, and as counts and percentages for categorical variables. A linear mixed-effects model (LMM) was used to compare the change in forced vital capacity (FVC) and diffusion capacity for carbon monoxide (DLCO) before and after LEF administration. The change in FVC and DLCO after 12 months of LEF initiation was estimated using the LMM, as the net difference in the observed change and the counterfactual change that would have been expected had treatment not been initiated. The actual and counterfactual trajectories of mean FVC and DLCO, centered on month of LEF initiation were plotted. The covariance used for the mixed effect model is ‘variance components’. The random term is ‘intercept’. In order to assess whether weight loss associated with prednisone dosage reduction was the cause of pulmonary function test (PFT) improvements, a Pearson correlation coefficient and linear regression model was constructed to check the relationship between weight change and FVC change. A subgroup analysis was performed on two groups stratified by the extent of fibrosis. A generalized mixed model with Poisson distribution was used to assess the change in corticosteroid dosage before and after LEF initiation. All the analyses were performed by using the SAS 9.4 for Linux (SAS, Cary, North Carolina). The level of statistical significance was set at *p* < 0.05 (two tailed).

## Results

A total of 40 patients with a multidisciplinary diagnosis of cHP who received treatment with LEF were identified. Their baseline characteristics are described in Table 1. The majority of the patients in this cohort were female (60%), Caucasian (92.5%) and either never or former smokers (35 and 62.5%, respectively). Sixteen patients (40%) had a surgical lung biopsy and a causative antigen was identified in 52.5%. Avian antigens (32.5% of the total cohort) were the most commonly identified antigen, when one could be found. The patients had already gone through antigen avoidance measures and still had persistent symptoms and physiologic changes. Patients had moderate restriction (FVC, 66.3 ± 19.2% of predicted) and moderately impaired gas exchange (DLCO, 51.4 ± 19.6% of predicted). The majority (32 patients, 80%) were on prednisone with a mean dose of 19.8 mg/day (± 10.8) at the time LEF was prescribed. LEF was the first steroid sparing agent used in 15 patients (37.5%). Other reasons for starting LEF were poor response to prior medication (13 patients, 32.5%) and adverse effects of previous treatment (8 patients, 20%). The maintenance dose of LEF was 20 mg/day in 35 patients (87.5%), and 10 mg/day in 5 patients (12.5%). None of our patients received a loading dose of LEF.

**Table 1:** Baseline characteristics

Characteristic	Total *N* = 40
Age (years) at initiation of leflunomide	61.5 ± 13.2
Race/Ethnicity	
•African American	2(5.0)
•Caucasian	37(92.5)
•Hispanic	1(2.5)
Gender	
•Female	24(60.0)
•Male	16(40.0)
Smoking history - Tobacco	
•Current smoker	1(2.5)
•Former	14(35.0)
•Never	25(62.5)
Is patient known to have antigen exposure?	
•Mold	5(12.5)
•Avian	13(32.5)
•Other	3(7.5)
•Unknown	19(47.5)
Surgical lung biopsy obtained	16(40.0)
Dose of Prednisone, mg	19.8 ± 10.8
PFTs at leflunomide initiation	
•FVC%	66.3 ± 19.2
•DLCO%	51.4 ± 19.6
CT or HRCT features (*N* = 28)^a^	
•Fibrosis > 20%	14 (50.0)
•Fibrosis ≤20%	4 (14.3)
•No fibrosis	10(35.7)
Reason for Leflunomide initiation	
•Adverse effect from prior medications	8(20.0)
•Poor response to prior medications	13(32.5)
•Started as 1st immunomodulatory agent	15(37.5)
•Other	4(10.0)

Statistics presented as Mean ± SD or N (% of total). *PFT* Pulmonary function test, *FVC* Forced vital capacity, *DLCO* Diffusing capacity for carbon monoxide, *CT* Computed tomography, *HRCT* High-resolution computed tomography

^a^*Imaging studies were reviewed for patients who were included for longitudinal analysis (N = 31). Three patients were excluded for missing data*

**Fig. 1:**
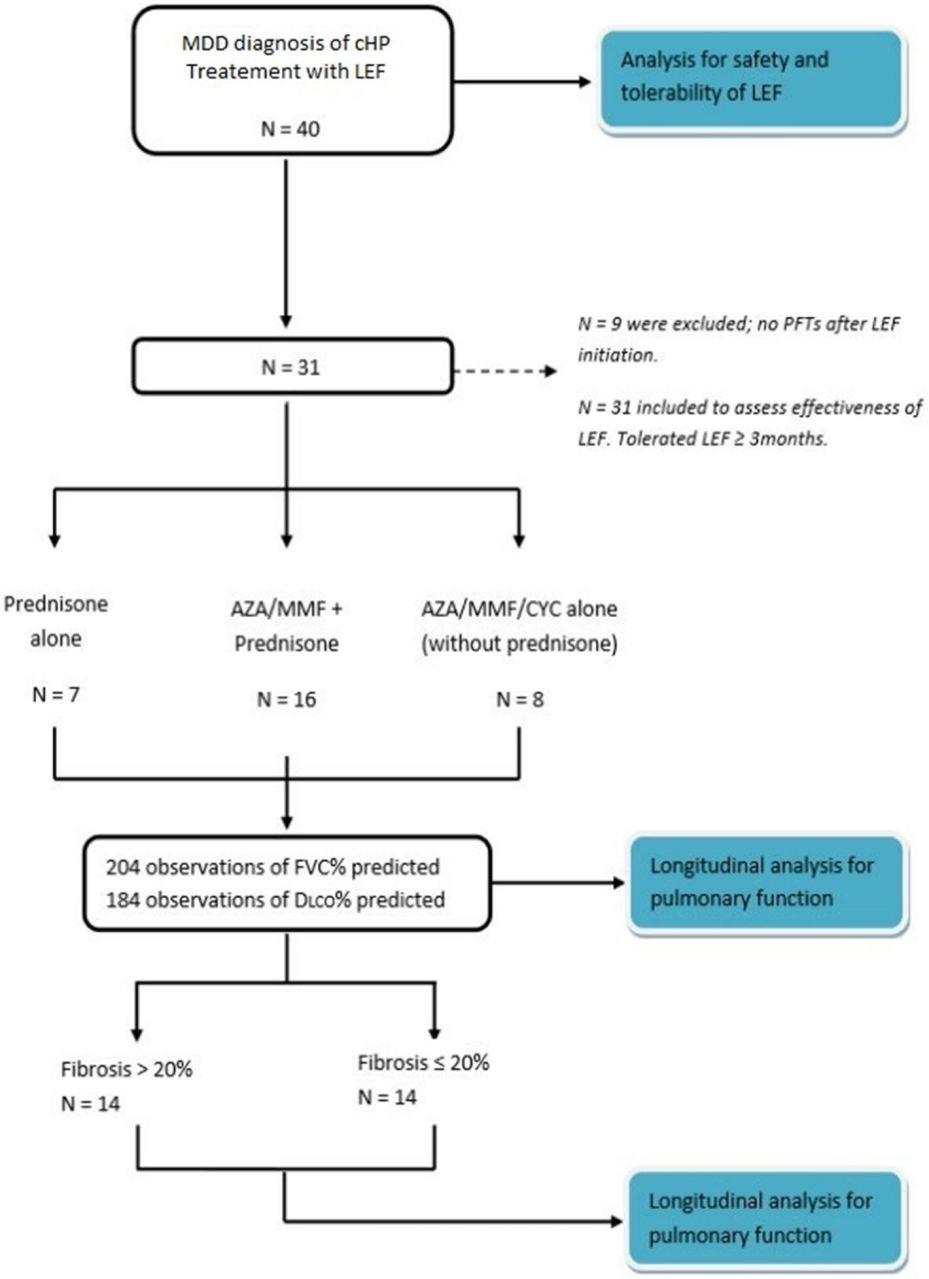
Chart flow of cohort formation. MDD: Multidisciplinary discussion, cHP: Chronic Hypersensitivity pneumonitis, LEF: Leflunomide, AZA: Azathioprine, MMF: Mycophenolate mofetil, CYC: Cyclophosphamide. * 3 patients were excluded from subgroup analysis for missing radiographic data

Of our 40 patients, 31 were included for longitudinal analysis of pulmonary function. Nine patients had to be excluded as LEF was discontinued before the first set of PFTs was performed, mainly because of adverse effects (Fig. 1). Of these 31 patients, 7 patients (22.6%) were receiving corticosteroid monotherapy. The other 24 patients (77.4%) were receiving other immunosuppressive agents (AZA *n* = 20, MMF *n* = 3, and Cyclophosphamide *n* = 1) with or without corticosteroids. Seven patients were on LEF for 3 to 6 months, and 24 patients were continued for over 6 months. The median duration of LEF therapy was 293 days (interquartile range 304), varying based on clinical response. A CT or HRCT within 12 months of LEF initiation was evaluated in the set of 31 patients; 3 had to be excluded as no imaging study was available during this period. Fourteen patients (50.0%) had more than 20% fibrosis on CT imaging, 4 patients (14.3%) had ≤20% fibrosis and no fibrosis was observed in 10 patients (35.7%).

**Fig. 2:**
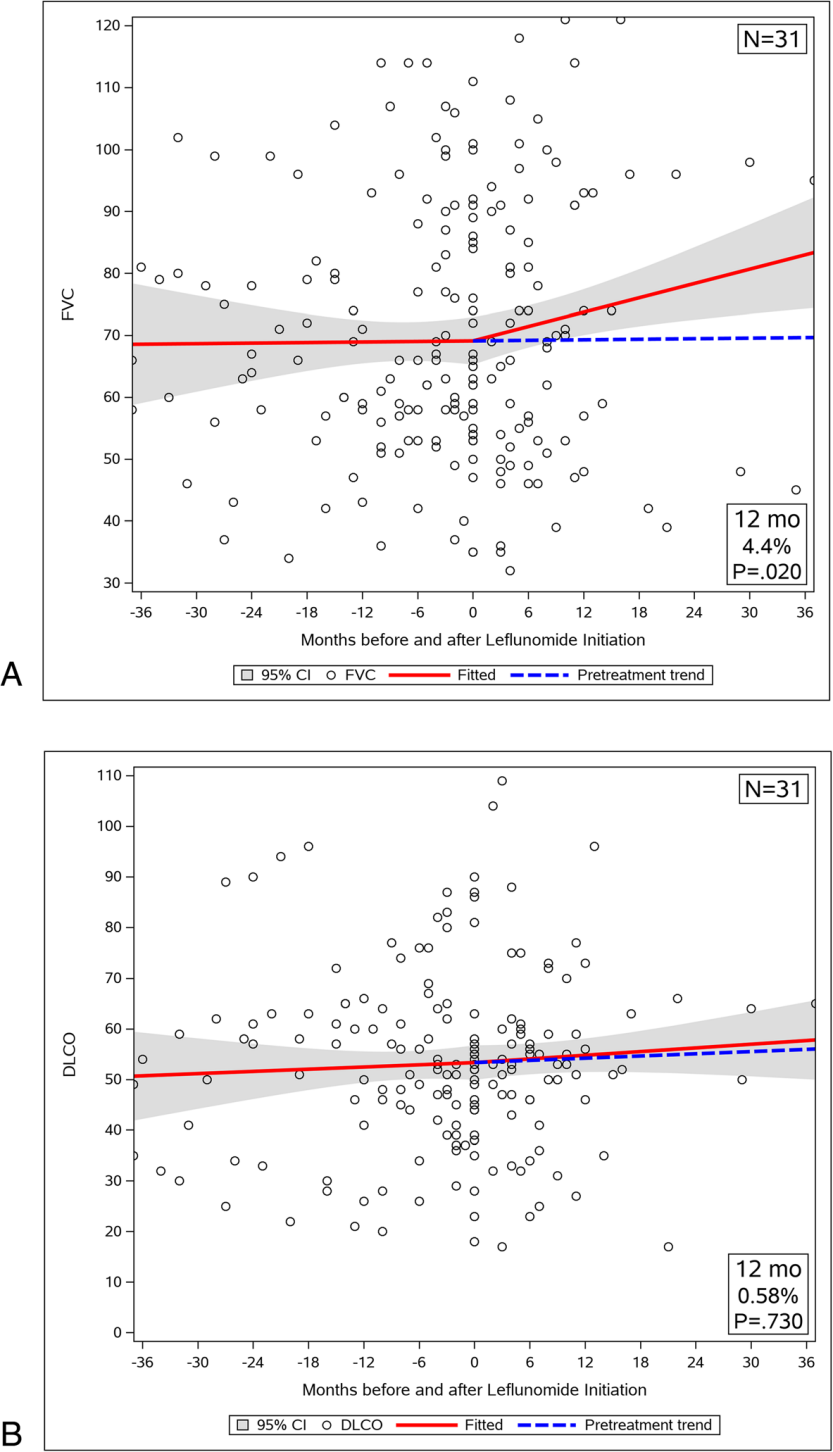
Trajectory of pulmonary function before and after LEF treatment in entire cohort. **a** The FVC trajectory improved with treatment from a rate of 0.18 ± 1.90% (SEM) predicted/year to 4.62 ± 1.65%predicted/year. FVC% predicted increased significantly at 12 months (mean increase 4.4% predicted; 95% CI, 0.7 to 8.5%; *p* = 0.020). **b** DLCO was already improving at 0.87 ± 1.70% (SEM) predicted/year and continued to improve at 1.45 ± 1.44%predicted/year with LEF therapy. DLCO% predicted did not change significantly after 12 months of LEF treatment (mean increase 0.58% predicted; 95% CI, − 2.7 to 3.9%; *p* = 0.730)

The cohort of 31 patients had a total 204 measurements of FVC and 184 measurements of DLCO before and after LEF initiation for longitudinal analysis. Prior to LEF initiation, the FVC trajectory was very gradually improving at a rate of 0.18 ± 1.90% (SEM) of predicted per year. Once LEF was started this trajectory began to improve markedly by 4.62 ± 1.65% of predicted per year. The difference between these slopes was statistically non-significant (Fig. 2a, *p* = 0.118). After 12 months of treatment however, the FVC had improved significantly by 4.4% of predicted, compared with what would have been expected had the pre-LEF trajectory continued (95% CI, 0.7 to 8.5%; *p* = 0.020). This FVC improvement was not associated with change in weight (*p* = NS). There was no significant correlation between the weight change and FVC% of predicted change after LEF initiation. Diffusion capacity also was already increasing at a rate of 0.87 ± 1.70% (SEM) of predicted per year prior to LEF initiation (Fig. 2b). It continued to trend towards an increase by 1.45 ± 1.44% of predicted per year once LEF was introduced. The DLCO had not changed significantly when measured after 12 months of LEF treatment (mean increase 0.58%; 95% CI, − 2.7 to 3.9%; *p* = 0.730).

**Fig. 3:**
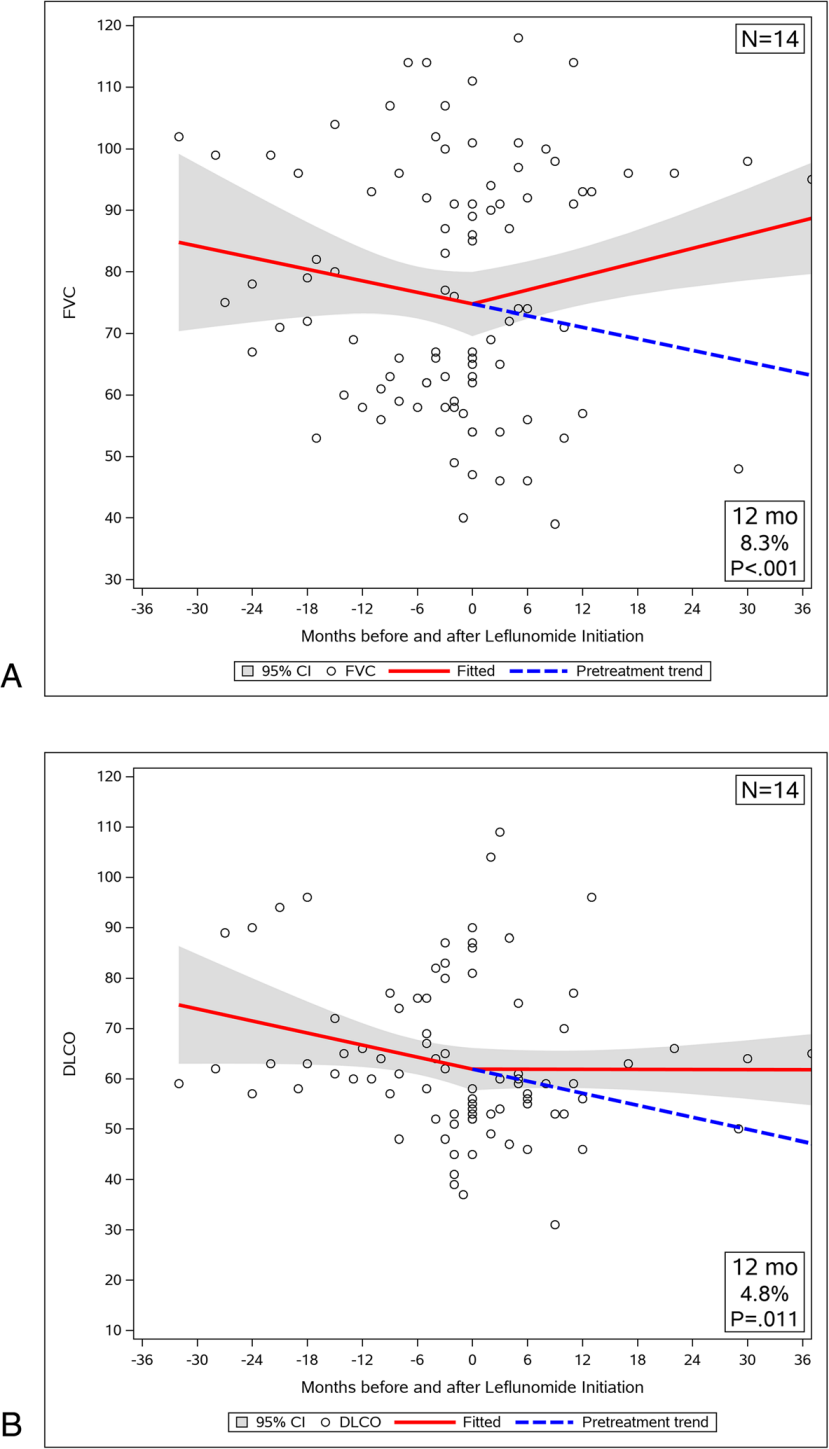
Trajectory of pulmonary function before and after LEF treatment in patients with fibrosis ≤20%. **a** FVC decline of 3.76 ± 3.18% (SEM) predicted/year was reversed to an increase of 4.52 ± 1.67% predicted/year with treatment. FVC% predicted increased significantly at 12 months (mean increase 8.3% predicted; 95% CI, 3.6 to 13.0%; *p* < 0.001). **b** The DLCO slope did not change significantly, from a DLCO of − 4.79 ± 2.57% (SEM) predicted/year to − 0.036 ± 1.31% predicted/year (*p* = 0.140), but DLCO% predicted increased significantly at 12 months (mean increase 4.8%predicted; 95% CI, 1.1 to 8.5%; *p* = 0.011)

**Fig. 4:**
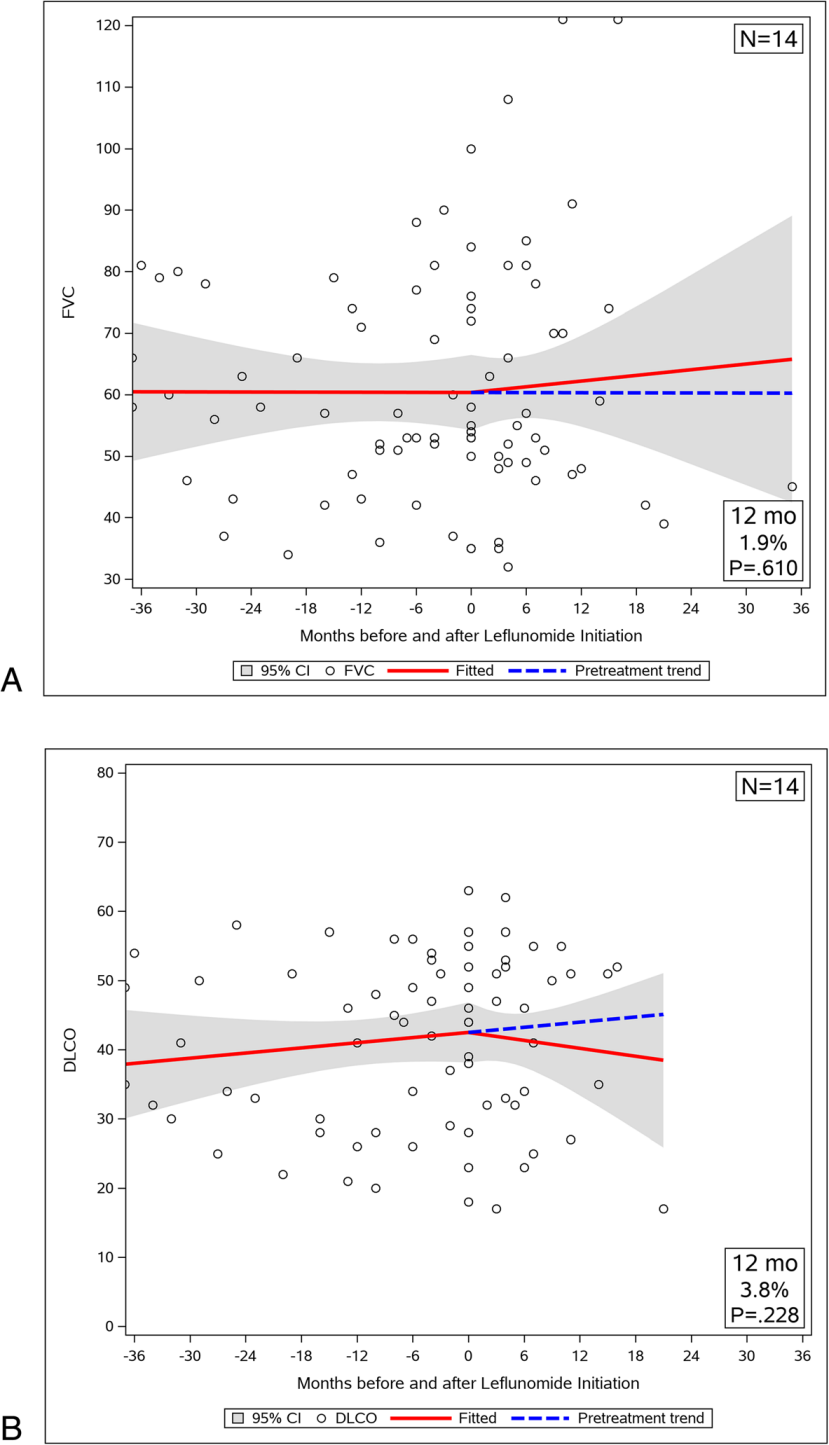
Trajectory of pulmonary function before and after LEF treatment in patients with fibrosis > 20%. **a** FVC decline of 0.037 ± 2.31% (SEM) predicted/year was reversed to an increase of 1.85 ± 4.54% predicted/year with treatment. However, FVC% predicted did not improve significantly at 12 months (mean increase 1.9%predicted; 95% CI, − 5.5 to 9.3%; *p* = 0.610). **b** The DLCO slope did not change significantly with treatment (*p* = 0.456). DLCO% predicted did not change significantly at 12 months (mean decrease 3.8%predicted; 95% CI, − 2.4 to 10.0%; *p* = 0.228)

The twenty-eight patients who had recent CT imaging available were divided in two subgroups according to the extent of radiographic fibrosis (≤ 20% and > 20%), and longitudinal analysis of pulmonary function was repeated. Fourteen patients (50%) were included in ≤20% fibrosis subgroup. In this subgroup the FVC trajectory was reversed significantly with LEF treatment from a decline of 3.76 ± 3.18% (SEM) of predicted per year to an improvement of 4.52 ± 1.67% of predicted per year (Fig. 3a, *p* = 0.04). There was no significant change in the DLCO slope before and after LEF therapy (Fig. 3b, *p* = 0.14), but the DLCO% of predicted increased significantly at 12 months (mean increase 4.8%; 95% CI, 1.1 to 8.5%; *p* = 0.011). Another 14 patients (50%) were assigned to the > 20% fibrosis subgroup. Their FVC trajectory improved after therapy from a decline of 0.037 ± 2.31% (SEM) of predicted per year to an improvement of 1.85 ± 4.54% of predicted per year, but the before and after slopes were not significantly different (Fig. 4a, *p* = 0.745). The diffusion capacity slopes for the subgroup with significant fibrosis did not change with LEF therapy either (Fig. 4b, *p* = 0.456).

**Fig. 5:**
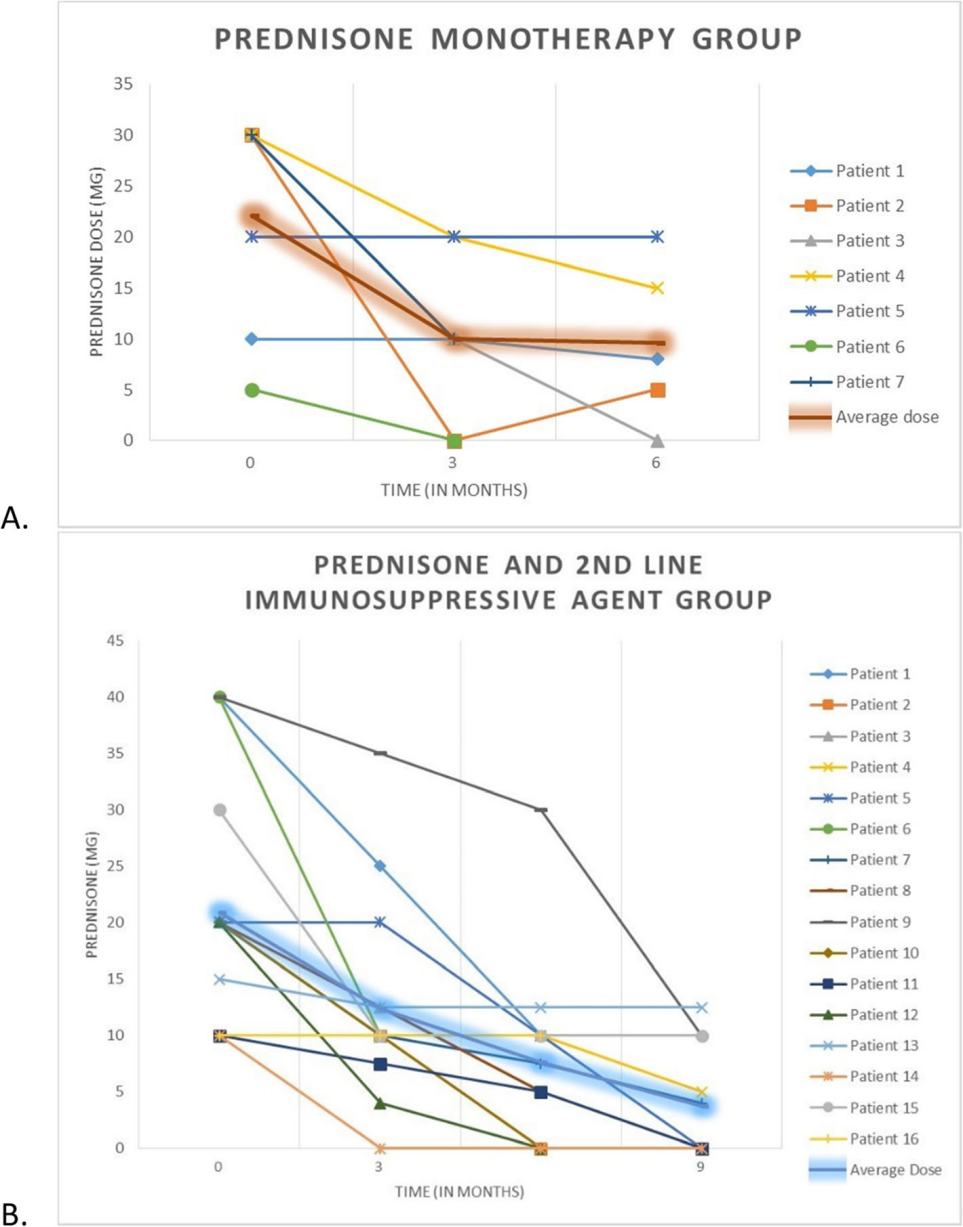
Prednisone dosages over time. LEF was initiated at time 0. Patients on prednisone alone (**a**) as well as those also receiving a second line immunosuppressant (**b**) were able to significantly decrease their dosage requirement

Twenty-three (74.1%) out of 31 patients were on prednisone at the time LEF was started. The mean dose of prednisone at LEF initiation was 21.7 mg/day (± 11.2) and there was a statistically significant decline in dose after LEF treatment (Fig. 5, *p* < 0.0001). Twelve of these 23 patients (52.1%) were weaned off prednisone within a year, and 7 patients (30.4%) were able to reduce their prednisone dose by ≥50% while on LEF therapy. The dose remained the same in 4 patients (17.4%) and none of the patients required an increase.

LEF therapy was associated with frequent adverse effects. Seventeen of 40 patients (42.5%) reported treatment related adverse events and 16 of these had to discontinue treatment. Interestingly, adverse events were more frequently reported in patients who were prescribed LEF as the first immunomodulatory agent (12 patients, 70.5%). Patients who were on other immunosuppressive agents prior to LEF therapy generally tolerated it well. Gastrointestinal symptoms including diarrhea (5 patients, 12.5%) and nausea (3 patients, 7.5%) were the most frequently reported, followed by elevated transaminases (4 patients, 10%), neuropathy (3 patients, 7.5%) and skin rash (3 patients, 7.5%) (Table 2).

**Table 2:** Side effects of Leflunomide

Side effect	*N* = 40
•Diarrhea	4(10.0)
•Nausea	3(7.5)
•Elevated transaminases	3(7.5)
•Neuropathy	3(7.5)
•Skin rash	3(7.5)
•Hair loss	1(2.5)
•Other	2(5.0)
•None	24(60.0)

Statistics presented as N(% of total). Some patients reported more than one side effect

## Discussion

To our knowledge, this is the first study evaluating the tolerability and effectiveness of LEF in patients with cHP. All the patients included had insidious onset of hypersensitivity pneumonitis, although only a subset had fibrosis. None of the included patients had rapid disease onset with an identifiable avoidable antigen. Thus, patients all fit into the category of chronic progressive or relapsing/remitting hypersensitivity pneumonitis. In keeping with the evolving trend to classify HP according to the degree of fibrosis and disease progression, we chose to divide our population into primarily fibrotic and non-fibrotic phenotypes.

The literature to guide management of patients with HP is unfortunately quite sparse. Three recent studies have advanced our understanding of the modern-day management of HP using corticosteroids and second line immunosuppressant agents. The recently published Belgian study of their experience with the use of prednisone in patients with HP is particularly instructive [10]. The authors divided patients into non-fibrotic and fibrotic subgroups based upon their CT appearance. They showed that corticosteroid therapy effectively reverses the declining FVC trajectory in patients with non-fibrotic HP but has no effect on disease progression in patients with fibrotic disease.

As T-cell mediated immunity plays a key role in the pathogenesis of HP, anti-lymphocyte agents such as AZA and MMF have been widely used in patients who have not responded to or not tolerated steroids. Morisset and colleagues showed that treatment of such patients with AZA and MMF was associated with significant improvement of gas exchange and reduction in corticosteroid dose [7]. In their study, 70 patients who were treated with either AZA or MMF had significant improvement in DLCO of 4.2% of predicted after 1 year of treatment. There was no significant change in FVC, which increased by just 0.5% of predicted (*p* = 0.46) at 1 year.

We now know that HP patients may respond differently to immunosuppressant therapy depending upon whether or not they have established fibrosis. It is difficult to categorize patients into mutually exclusive fibrotic and non-fibrotic subtypes. There is bound to be some overlap, depending upon the extent of fibrosis. Presumably patients with early fibrosis have a significant component of ongoing inflammation that may benefit from immunomodulation. Consequently, unlike De Sadeleer and colleagues who placed patients with any fibrosis into the “fibrotic HP” category [10], we arbitrarily chose 20% fibrosis on CT as our threshold to categorize patients.

There was a noticeable difference in the pre-treatment pulmonary function trajectory when we divided patients into groups based on presence or absence of established fibrosis (Figs. 3 and 4). Despite treatment with immunosuppressive agents of known efficacy, our < 20% fibrosis group clearly continued to decline, whilst those with established fibrosis appeared to have stabilized, albeit at a greater degree of impairment. This suggests that the former group had persistent inflammation because of undertreatment or inability to tolerate treatment, which could potentially progress to established fibrosis and plateaued pulmonary function.

LEF had been added to our cohort based on the hypothesis that there may be persistent inflammation and the patients’ desire to augment therapy with the goal of obtaining additional symptomatic and physiologic improvement. In some cases, LEF was added because the patient was tolerating prior therapy poorly. We too found that LEF did not have any effect on pulmonary function in patients with fibrotic HP. Patients without established fibrosis however, responded quite well to therapy.

Our study demonstrates that the addition of LEF to prednisone and/or second line immunosuppressant therapy can lead to further gains in pulmonary function for treated patients. This manifested as a significantly improved FVC in our HP patients after 12 months. As the majority of our patients were already on AZA/MMF, their DLCO was improving even prior to the introduction of LEF. This pretreatment trend continued once AZA/MMF was switched to LEF. Change in body weight is known to be associated with change in FVC [11]. We confirmed that the improvement in FVC in our patients was independent of any change in their weight.

Adegunsoye and colleagues did not find a significant change in pulmonary function with immunosuppressant therapy compared with prednisone alone in their chronic HP patients [6]. They did however note that treatment related adverse effects were far less frequent with use of AZA or MMF. It is well known that long-term corticosteroid use is associated with cumulative toxicity. A systematic review of recent literature confirms that steroid adverse effects with such treatment remain associated with significant clinical and economic burden [12]. These include a greater than 30% increase in hypertension, an up to 4-fold increase in hyperglycemia and type 2 diabetes, and a 20–30% prevalence of fractures. Two thirds of our patients, both fibrotic and non-fibrotic, were on corticosteroid therapy before LEF was started. The drug had a significant steroid sparing effect, allowing half of these patients to eliminate prednisone entirely.

Our data show that LEF therapy is associated with significant improvements in pulmonary function and a steroid-sparing effect. This effect is most pronounced in patients without established/significant fibrosis (< 20%). Fifty two percent of the patients treated with LEF in our study were able to eliminate prednisone entirely, and another 30% were able to decrease their dose at least by half. Based on these findings, LEF appears to suppress active inflammation and possibly progression to/of fibrosis in cHP. Notably, the majority of our cohort had already failed other agents.

The exact mechanism whereby LEF arrests and reverses declining pulmonary function in HP patients remains unclear. It also remains unknown whether or not the favorable effect of LEF on pulmonary function translates into improved quality of life or decreased mortality.

Our enthusiasm for LEF must be tempered by its frequent adverse effects. In our study these led a substantial 40% of patients to discontinue the drug. This rate of medication discontinuation is higher than previous reports of its use in patients with RA or pulmonary sarcoidosis, which was 19–22% [13–15]. We found that in our cohort, LEF was most likely to be discontinued when it was used as the first steroid sparing agent. Under the circumstances, patients and physicians were more inclined to switch to alternative agents with greater literature support if they were to experience even mild adverse effects.

Our study has limitations. This was the reported experience at a single center, with a modest number of patients. Its retrospective nature and the lack of a control group limit the strength of conclusions one can draw about any particular intervention. A referral bias is inherent given that the study was of a population presenting to a tertiary care center. Our study is strengthened by the well characterized nature of the patients, all diagnosed by multidisciplinary discussion. It is bolstered by how well our HP cohort physiologically mirrors other experiences [7]. Our work accords with the finding that MMF and AZA improve the diffusion capacity in patients with HP, as well as the improvement in FVC conferred by corticosteroid therapy. The use of linear mixed modelling to compare the effect of numerous pulmonary function data points before and after LEF initiation is statistically more robust than comparing a set of mean pre and post values. All of our patients were on systemic corticosteroids and/or AZA and MMF prior to being started on LEF. This is reflected in their gently improving FVC and DLCO trajectories even prior to initiating LEF [7, 10]. It is gratifying to report the further increase in pulmonary function our patients experienced with the addition of LEF, particularly in those without established fibrosis.

## Conclusions

Our study suggests that LEF is an effective steroid sparing immunomodulatory drug for patients with cHP, which was demonstrated by improvement in pulmonary function and reduction of corticosteroid dosage. No previously unknown adverse effects were reported. Leflunomide therapy was most beneficial to patients without established fibrosis. Prospective randomized controlled trials are needed to further evaluate the role of LEF in the management of HP.

## Data Availability

The datasets generated and/or analyzed during the current study are available in the Mendeley repository, 10.17632/sh25p653p9.1

## Abbreviations

cHP: Chronic hypersensitivity pneumonitis
ILD: Interstitial lung disease
AZA: Azathioprine
MMF: Mycophenolate mofetil
LEF: Leflunomide
CYC: Cyclophosphamide
RA: Rheumatoid arthritis
LMM: Linear mixed effects model
FVC: Forced Vital capacity
DLCO: Diffusion capacity for carbon monoxide
PFT: Pulmonary function test
SEM: Standard error of mean
MDD: Multidisciplinary discussion
